# Fatigability: An Early Marker of Diminished Renal Function?

**DOI:** 10.1093/geroni/igab046.797

**Published:** 2021-12-17

**Authors:** Eleanor Simonsick, Ann Moore, Michelle Shardell, Pei-Lun Kuo, Ajoy Karikkineth, Luigi Ferrucci

**Affiliations:** 1 National Instute on Aging/NIH, Baltimore, Maryland, United States; 2 National Institute on Aging, Baltimore, Maryland, United States; 3 University of Maryland School of Medicine, Baltimore, Maryland, United States; 4 National Institute on Aging, National Institute on Aging, Maryland, United States

## Abstract

Renal function declines markedly with age due to normal aging and/or disease processes and impacts multiple systems. Diminished renal function may manifest as low exercise tolerance and fatigue threshold. Using data on 951 well-functioning (usual gait speed >.67m/s and no difficulty walking ¼ mile) men and women (51%) aged 60-89 years in the Baltimore Longitudinal Study of Aging, we evaluated the cross-sectional association between perceived fatigability (Rating Perceived Exertion after 5-minute treadmill walk at 1.5mph) categorized as 6-7, 8-9, 10-11 and 12+ and GFR using Cockcroft-Gault. For each fatigability increment, likelihood of suboptimal (GFR=75-89, 21%), diminished (GFR=60-74, 26%) and poor renal function (GFR=15-59, 30%) relative to GFR≥90 was respectively OR(95%CI)p-value 1.51(1.16-1.96).002, 1.38(1.04-1.83).027 and 1.68(1.22-2.31).002 adjusted for demographics, weight, height, smoking, exercise and anemia. Findings were similar for men and women. Perceived fatigability may facilitate identification of apparently well-functioning older adults on the precipice of suboptimal to poor renal function.

